# Formation of Magnetic Anisotropy by Lithography

**DOI:** 10.1038/srep26709

**Published:** 2016-05-24

**Authors:** Si Nyeon Kim, Yoon Jae Nam, Yang Doo Kim, Jun Woo Choi, Heon Lee, Sang Ho Lim

**Affiliations:** 1 Department of Materials Science and Engineering, Korea University, Seoul 02841, Korea; 2 Research and Development Division, SK Hynix Semiconductor Inc., Icheon 17336, Korea; 3 Center for Spintronics Research, Korea Institute of Science and Technology, Seoul 02792, Korea

## Abstract

Artificial interface anisotropy is demonstrated in alternating Co/Pt and Co/Pd stripe patterns, providing a means of forming magnetic anisotropy using lithography. In-plane hysteresis loops measured along two principal directions are explained in depth by two competing shape and interface anisotropies, thus confirming the formation of interface anisotropy at the Co/Pt and Co/Pd interfaces of the stripe patterns. The measured interface anisotropy energies, which are in the range of 0.2–0.3 erg/cm^2^ for both stripes, are smaller than those observed in conventional multilayers, indicating a decrease in smoothness of the interfaces when formed by lithography. The demonstration of interface anisotropy in the Co/Pt and Co/Pd stripe patterns is of significant practical importance, because this setup makes it possible to form anisotropy using lithography and to modulate its strength by controlling the pattern width. Furthermore, this makes it possible to form more complex interface anisotropy by fabricating two-dimensional patterns. These artificial anisotropies are expected to open up new device applications such as multilevel bits using in-plane magnetoresistive thin-film structures.

Interface effects are known to be responsible for the perpendicular magnetic anisotropy (PMA) exhibited by [Co/Pt] and [Co/Pd] multilayers^1^,^2^,^3^. In order for these multilayers to exhibit PMA however, the intrinsic interface anisotropy must be strong enough to overcome a large demagnetizing field of ~4π*M*_s_ (where *M*_s_ is saturation magnetization). This indicates that in conventional multilayers, the intrinsic PMA is “screened” by the demagnetizing field, resulting in an effective PMA that is substantially reduced. This screening effect can be avoided if the Co/Pt and Co/Pd interfaces are formed along the film plane. One way to realize this is by forming Co/Pt and Co/Pd stripe patterns, which can be easily fabricated using lithography such as photolithography and nanoimprint lithography. The roughness of the Co/Pt and Co/Pd interfaces in the stripe patterns is expected to be poorer than that in conventional [Co/Pt] and [Co/Pd] multilayers, which are formed during deposition. Because the interface anisotropy is sensitive to the smoothness of the interface^4^,^5^,^6^,^7^, the anisotropy formed in the Co/Pt and Co/Pd stripe patterns is expected to be small. In spite of this, the formation of interface anisotropy in Co/Pt and Co/Pd stripe patterns can be of significant importance, because magnetic anisotropy can be formed using lithography. Currently, the anisotropy in magnetic thin films is typically induced by field-sputtering or field-annealing (so called induced anisotropy)^8^,^9^. The induced anisotropy thus formed is uniaxial and its direction is parallel to the direction of the applied magnetic field during sputtering or annealing. Owing to the difficulty in localizing the applied magnetic field, it is not easy to control the direction of induced anisotropy on small scales. However, this problem can easily be solved in Co/Pt and Co/Pd stripe patterns if the interface anisotropy is created at the Co/Pt and Co/Pd interfaces, as complex Co/Pd patterns can easily be fabricated using lithography. In this work, Co/Pt and Co/Pd stripe patterns of three different widths are fabricated using lithography and their magnetic properties are measured in order to characterize their anisotropy properties.

## Results

### 50 and 20 μm widths stripe patterns formed by photolithography

**Figure 1:**
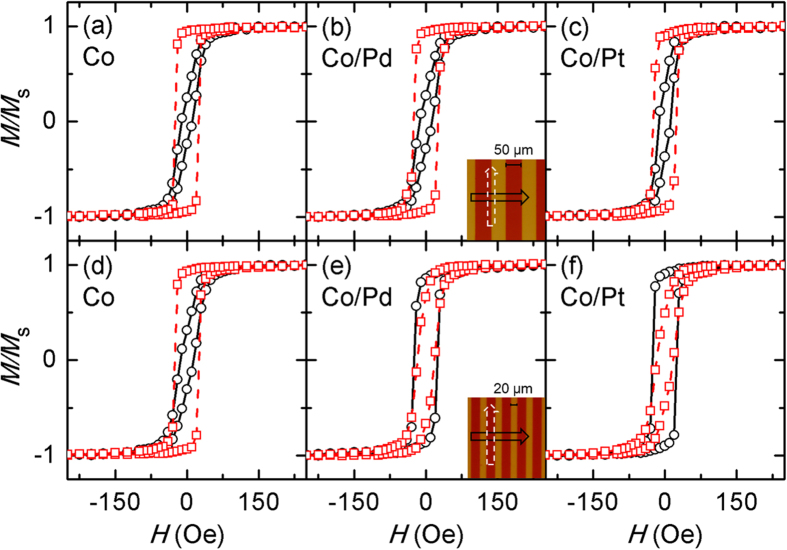
In-plane hysteresis loops of the Co stripes, Co/Pd and Co/Pt stripe patterns with two different widths along two principal directions: the width direction (solid line and circles) and the length direction (dashed line and squares). The results correspond to the samples (**a–c**) 50 and (**d–f**) 20 μm widths stripe patterns. An optical microscopy image of the patterned structure is shown in the insets of (**b,e**).

Figure 1(a–f) show the in-plane *M*−*H* (where *M* and *H* are the magnetization and applied magnetic field, respectively) hysteresis loops, normalized with respect to *M*_s_, along the length (dashed lines and squares) and width directions (solid lines and circles). The upper three panels are for the patterns 50 μm in width, whereas the lower ones are for those 20 μm in width. The left panels show the results for the Co pattern only, whereas the middle and right panels show the results for the Co/Pd and Co/Pt stripes, respectively. Insets in Fig. 1(b,e) show optical microscopy images (top-down views) of the respective stripe patterns. For the 50 μm width patterns as shown in Fig. 1(a–c), the remanence ratios along the length direction are higher than those along the width direction for all cases of Co, Co/Pd and Co/Pt stripes. These results indicate that the shape anisotropy is dominant over the interface anisotropy. In fact, the loops of the Co/Pd and Co/Pt stripe patterns have a shape similar to those of the Co pattern. Here, it is worth noting the combination effects of two uniaxial anisotropies; when two uniaxial anisotropies cross at right angles (90°), which is the case here, the combined anisotropy points toward the direction of the stronger anisotropy, with its strength given by the difference between the two^10^,^11^,^12^.

For the 20 μm width patterns as shown in Fig. 1(d–f), a different situation arises. In this case, the more favorable axis is along the length direction for the Co pattern due to the sole existence of the shape anisotropy (Fig. 1(d)), but it shifts to the width direction in the cases of the Co/Pd and Co/Pt stripe patterns (Fig. 1(e,f)), with this tendency being more stronger in the latter Co/Pt patterns. Obviously, this switch between axes is due to the stronger contribution of the interface anisotropy as the width of the stripes becomes narrower. Two effects are expected to arise from the reduction of the stripe width from 50 to 20 μm. One is the increase of the shape anisotropy due to the increase in the aspect ratio of the Co stripes. This effect, however, appears to be negligible, as evidenced by the fact that the magnetic properties of the Co only patterns are similar to each other (Fig. 1(a,d)). One possible explanation is that the aspect ratios of both 20 and 50 μm width patterns, calculated to be 450 and 180 respectively, are high enough to be in the saturation regime^13^; at small aspect ratios, the shape anisotropy continuously increases with increasing aspect ratio and then saturates after reaching a certain limit. The other possibility is related to the formation of multi-domains, which is confirmed by magneto-optical Kerr effect (MOKE) microscopy. In spite of this, we carried out a more quantitative analysis to find the difference in the shape anisotropy. A well-known method known as the alternating field method was used, which involves progressively reducing the applied magnetic fields until the hysteresis reached a minimum value. The slope at small magnetic fields is then extrapolated to *M*_s_ to determine the anisotropy field^14^,^15^. However, it was not possible to obtain reliable results from the method, because the region showing the non-hysteresis is narrow and hence the extrapolation to *M*_s_ is very large. The other effect is the increase in the interface anisotropy due to the increase in the number of Co/Pt and Co/Pd interfaces. From the entire lateral dimensions of the sample (9 mm × 9 mm), except for losses of the edges coated with a thick photoresist (PR) during spin coating, the number of the interfaces can be calculated in a straightforward manner. This value is 90 for the wide stripe patterns and 225 for the narrow stripe patterns.

Although the results for the 20 μm width patterns as shown in Fig. 1(e,f) clearly indicate that the interface anisotropy is dominant over the shape anisotropy, a quantitative analysis on the strength of the two anisotropies is difficult to make due to the hysteresis of the hard-axis loops. Because the stripes are all parallel to each other, this hysteresis is due to the formation of multi-domains, and possibly the distribution of the interface anisotropy. Even with this information, it will be of some value to estimate the level of the interface anisotropy formed in these stripes. A simple single-domain calculation gives a shape anisotropy value of 14 Oe, which is an upper limit and can likely be reduced by as much as half through the formation of multi-domains. Considering that the interface anisotropy is only slightly stronger than the shape anisotropy in the cases of both the Co/Pd and Co/Pt stripes, the interface anisotropy formed is of this level.

### 382 nm width stripe patterns formed by nanoimprint lithography

**Figure 2:**
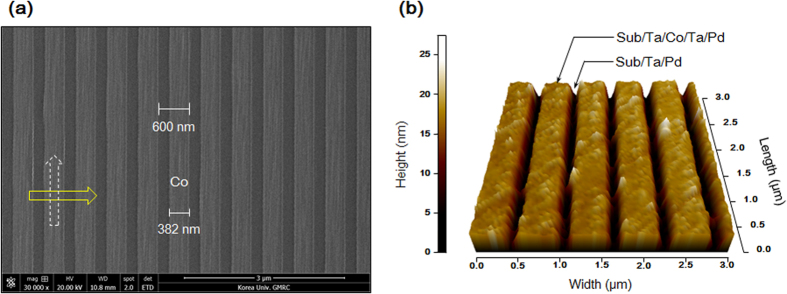
Top view of the patterned sample surfaces fabricated on the Ta (5 nm) buffered Si/SiO_2_ substrate. (**a**) SEM image of the surface of Co stripes. (**b**) AFM image of the surface of Co/Pd stripe patterns. The AFM image corresponds to a 3 × 3 μm^2^ sample area, and the black to white color contrast scales height from 0 to 27 nm.

For a more quantitative analysis on the strength of the two anisotropies, samples with narrower patterns (382 nm width) were fabricated using nanoimprint lithography. In addition to the Si/SiO_2_ substrate used for the wide stripe patterns, a Ta (5 nm) buffered substrate (i.e. Si/SiO_2_/Ta) was also used to create better adhesion with the substrate. Figure 2(a) shows a scanning electron microscopy (SEM) image of the Co patterns on the Ta buffered substrate, whereas Fig. 2(b) displays an atomic force microscopy (AFM) image of Co/Pd stripe patterns on the same substrate. These images indicate that the patterns are regularly arrayed, and furthermore the deposition of Pd on well-defined Co stripes is neat and free of defects. The difference in the thickness between Co in the patterned area and Pd in the unpatterned area is measured to be precisely 10 nm. Although the black to white color contrast scales from 0 to 27 nm in height, these results are reasonable considering the oxidation of Ta surface and the resolution limits inherent to AFM. High-resolution transmission electron microscopy (HR-TEM) experiments were performed to examine the Co/Pd interfaces of the 382 nm width Co/Pd stripe patterns on the Ta (5 nm) buffered substrate. Cross-sectional scanning transmission electron microscopy (STEM) images and energy disperse X-ray spectroscopy (EDS) line profiles (results not shown) indicate that the Co/Pd interfaces are clearly formed.

**Figure 3:**
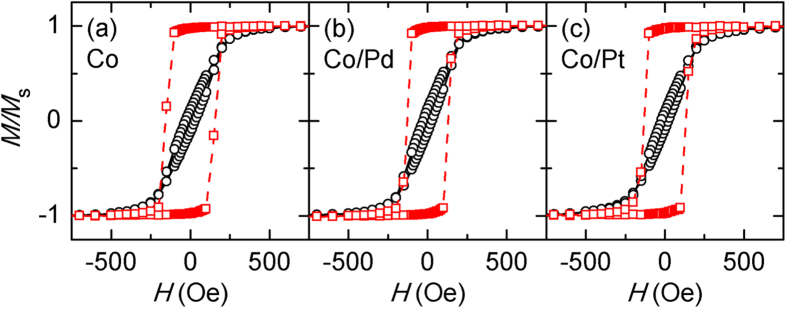
In-plane hysteresis loops of the 382 nm width stripe patterns along the width direction (solid line and circles) and the length direction (dashed line and squares). The results are for the samples (**a**) Co stripes, (**b**) Co/Pd stripe patterns and (**c**) Co/Pt stripe patterns on the Si/SiO_2_ substrate.

Results for the narrow stripes on the Si/SiO_2_ substrate are displayed in Fig. 3(a–c), similar to those shown in Fig. 1. As expected, the hysteresis along the hard axis is significantly reduced, indicating that the samples are close to a single-domain state and hence magnetization along this direction mainly occurs by coherent rotation. MOKE microscopy images (results not shown), taken at several important steps of the magnetization process, confirm these two features. Specifically, magnetic domains start to reverse nearly at random locations and the region of reversed domains increases with the increase of *H*. An important feature to note during this magnetization process is that the reversed regions are not close to the existing ones, indicating that magnetization occurs not by domain-wall motion but by magnetization rotation.

Although the number of Co/Pt and Co/Pd interfaces in the narrow stripes increases by a factor of 67 over what is observed in the 20 μm width stripes, the magnetic properties are dominated by the shape anisotropy, which is likely caused by the magnetic configuration being close to a single-domain state. The results in Fig. 3(a) for the Co only patterns can be used to calculate their shape anisotropy. However, due to the existence of the (albeit very small) hysteresis along the hard axis, the results in their present form cannot be used to extract the shape anisotropy. In order to solve this problem, the same alternating field method, used previously for the 50 and 20 μm widths Co stripes, was employed to determine the anisotropy for the 382 nm width stripe patterns. The shape anisotropy of Co stripes as shown in Fig. 3(a) is estimated to be 261 Oe. For Co/Pd and Co/Pt stripes, the results of which are shown in Fig. 3(b,c), an additional interface anisotropy should exist together with the shape anisotropy, thus reducing the overall anisotropy of the stripe patterns. The overall anisotropies of the Co/Pd and Co/Pt stripes are estimated to be 237 and 244 Oe, respectively. The interface anisotropies, which can be obtained from the differences between the shape anisotropy in the Co only stripes and the overall anisotropy in the Co/Pd and Co/Pt stripes, are estimated to be 24 and 17 Oe respectively. A similar set of results were obtained from independent experiments using the Ta-buffered substrate. The same procedure gives interface anisotropies of 20 and 14 Oe for the Co/Pd and Co/Pt stripes, respectively.

## Discussion

The strength of the interface anisotropy observed for the narrow-width stripes is of a similar magnitude to that of the induced anisotropy in many previously studied magnetic systems^16^,^17^,^18^. For the 382 nm width stripes, where reasonably accurate values of interface anisotropy can be obtained, the interface anisotropy energy density is in the range of 1.0 × 10^4^ to 1.7 × 10^4^ erg/cm^3^ (or 0.2–0.3 erg/cm^2^ in terms of the interface anisotropy energy per unit area). When these values are compared with those reported in the literature for conventional [Co/Pd] and [Co/Pt] multilayers, a review article by Johnson *et al*.^3^ (where a comprehensive summary on the interface anisotropy energy is provided) reports values in the range of 0.2–0.97 erg/cm^2^ for conventional [Co/Pd] and [Co/Pt] multilayers. These values strongly depend on the type of substrates and buffer layers and the crystallographic plane^3^. More recently, Lee *et al*. reported similar values in the range of 0.2–1.14 erg/cm^2^ for [Pt/Co] multilayers with an inverted structure depending on the Co thickness^19^. The results for the interface anisotropy energy observed from the present Co/Pd and Co/Pt stripes are within the range reported for the conventional multilayers, but are located close to the lower boundary. This indicates that the roughness of the interfaces in the present stripe patterns is poorer than that in conventional multilayers where the interfaces are formed under highly controlled conditions, and a measure of forming suitable interface textures for high interface anisotropy is usually taken during deposition. It is therefore important to develop new lithographic processes, which enable to improve the roughness of the interfaces in the stripe patterns. One possible option can be to use low-energy proton irradiation, which was recently used to obtain strong perpendicular magnetic anisotropy at the Co/Pd interfaces^20^.

The demonstration of interface anisotropy in the Co/Pt and Co/Pd stripe patterns is of significant practical importance, because this setup makes it possible to form anisotropy using lithography and to modulate its strength by controlling the pattern width (and, more precisely, the interface density). Furthermore, this makes it possible to form more complex interface anisotropy by fabricating two-dimensional patterns; for example, a four-fold anisotropy can be formed with a mosaic pattern and even a six-fold anisotropy can be achieved with a hexagonal pattern. These four- or six-fold “artificial” anisotropies can be incorporated into in-plane magnetic tunnel junctions to realize multi-bits. Another interesting possibility is the formation of radial anisotropy in Co nanoparticles coated with Pd or Pt.

## Methods

### Fabrication steps of both photolithography and nanoimprint lithography

**Figure 4:**
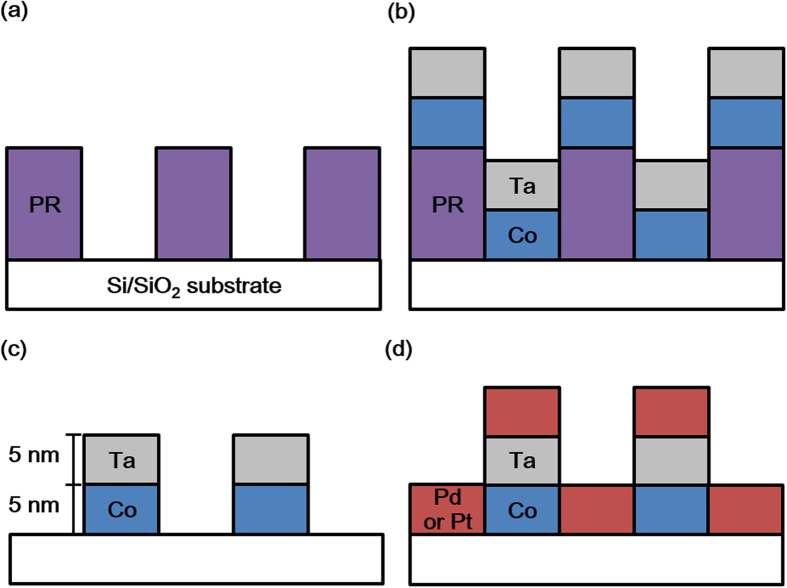
Important steps in the fabrication of micrometer- and nanometer-width alternating Co/Pd stripe patterns. (**a**) PR patterns on a thermally oxidized Si substrate. (**b**) The deposition of Co and Ta layers on the PR patterns. (**c**) The lift-off process to remove the PR patterns. (**d**) The deposition of Pd layer to form the final stripe patterns.

The Co/Pt and Co/Pd stripe patterns were fabricated using a conventional lithography technique and a schematic of the fabrication steps is illustrated in Fig. 4(a–d). Stripe patterns with widths on the order of 10^1^ μm can be fabricated using photolithography, whereas those with widths on the order of 10^2^ nm can be formed by nanoimprint lithography. Photolithography was carried out to fabricate stripe patterns with 50 and 20 μm widths. The first step was to form PR patterns on a thermally oxidized Si substrate with lift-off-layer (LOL) solvent and AZ 4210 positive PR (Fig. 4(a)). The second step was to deposit layers of Co and Ta using an ultra-high-vacuum (UHV) dc magnetron sputter, forming layers of 5 nm thickness (Fig. 4(b)). Ta was used as a capping layer to prevent the oxidation of Co. The PR patterns were then removed through a lift-off process using an AZ 400K developer (Fig. 4(c)), followed by deposition of the Pd or Pt layer with a thickness of 5 nm, resulting in the final structure (Fig. 4(d)). Just prior to the deposition of Pd or Pt, the Co oxide formed at the surface of the Co stripes during the lithography process was removed by 5 min of plasma exposure. Unlike Co, no capping layer was deposited on top of Pd or Pt because of the chemically inert nature of these metals. Nanoimprint lithography was conducted to fabricate stripe patterns 382 nm in width. A similar process was carried out for the case using photolithography, although some details differ. Two different substrates of a thermally oxidized Si and a 5 nm thick Ta buffered Si/SiO_2_ substrate were used. The first step was to form PR patterns on the substrate using an LOL solvent and hydrogen silsesquioxane (HSQ). Contrary to the process of using masks and UV exposure in photolithography, a stamp with a 300 nm width was used to form PR patterns. A reactive ion etching (RIE) process was then carried out to remove the residual PR in the patterned area. During the RIE process, the etchant gas CF_4_/O_2_ damages the surface of the Si/SiO_2_ substrate owing to the reaction between SiO_2_ and CF_4_. Unlike the photolithography method, the Co and Ta layers were each deposited at 5 nm thickness using an electron-beam (e-beam) evaporator. This is because the e-beam evaporation, due to the thin film growth without adhesion between the deposits and existing patterned PR, works better with the subsequent lift-off process than the sputtering. Considering that an e-beam evaporator has small shadowing effects than a sputter, these undesirable effects can be minimized during the deposition of the Co and Ta layers with the use of the e-beam evaporator. Both the LOL and upper HSQ layers were developed using dimethylformaldehyde (DMF). The structure was then exposed to plasma for 5 min, followed by the deposition of a 5 nm-thick Pd layer with the UHV dc magnetron sputter.

### Deposition methods

All layers were deposited at thicknesses of 5 nm using a UHV dc magnetron sputter or an e-beam evaporator. For sputtering, the base pressure was 7 × 10^–8^ torr and the Ar pressure during deposition was 2 × 10^–3^ torr. For e-beam evaporation, the base pressure was 7.5 × 10^–9^ torr. The deposition rates of the layers were measured in advance, and this information was used to control the thickness by choosing an appropriate sputtering time. In the determination of the deposition rate, the thickness was measured using a surface profiler. The deposition rates were adjusted to ~0.03 nm/s for sputtering and ~0.1 nm/s for e-beam evaporation by regulating the power during deposition. The Co/Pt and Co/Pd stripe patterns were formed over a 10 mm × 10 mm region.

### Magnetic and microstructural characterization

The magnetic anisotropy properties were mainly characterized by measuring *M*−*H* hysteresis loops under in-plane magnetic fields with a vibrating sample magnetometer (VSM). The loops were measured along two principal directions, the length (or longitudinal) direction and the width (or transverse) direction. Magnetic domains were observed using a MOKE microscope with a spatial resolution limit of 1 μm. The microstructure of the stripe patterns was examined by HR-TEM and the composition was analyzed using EDS line profiling.

## Additional Information

**How to cite this article**: Kim, S. N. *et al*. Formation of Magnetic Anisotropy by Lithography. *Sci. Rep.* **6**, 26709; doi: 10.1038/srep26709 (2016).
